# Voices off: Stanley Milgram’s cyranoids in historical context

**DOI:** 10.1177/0952695119867021

**Published:** 2019-09-24

**Authors:** Marcia Holmes, Daniel Pick

**Affiliations:** Birkbeck, University of London, UK; Birkbeck, University of London, UK

**Keywords:** 1970s, Cold War, historiography, history of psychology, social psychology

## Abstract

This article revisits a forgotten, late project by the social psychologist Stanley Milgram: the ‘cyranoid’ studies he conducted from 1977 to 1984. These investigations, inspired by the play *Cyrano de Bergerac*, explored how individuals often fail to notice when others do not speak their own thoughts, but instead relay messages from a hidden source. We situate these experiments amidst the intellectual, cultural, and political concerns of late Cold War America, and show how Milgram’s studies pulled together a variety of ideas, anxieties, and interests that were prevalent at that time and have returned in new guises since. In discussing the cyranoid project’s background and afterlife, we argue that its strikingly equivocal quality has lent itself to multiple reinterpretations by historians, psychologists, performers, artists, and others. Our purpose is neither to champion Milgram’s work nor to amplify the critiques already made of his methods. Rather, it is to consider the uncertain, allusive, and elusive aspects of the cyranoid project, and to seek to place that project in context, whilst asking where ‘context’ might end. We show how the experiments’ range of meanings, in different temporal registers, far exceeded the explanatory rubric that Milgram and his intellectual critics provided at that time, and ponder the risk for the historian of making anachronistic or teleological assumptions. In short, we argue, cyranoids invite our open-ended exploration of ‘voices offstage’ in social and psychological relations, and offer a useful tool for thinking about historical context and the nature of historical interpretations.

It is 1981. An avuncular Stanley Milgram – the famed American social psychologist, 48 years old but already in declining health – sits in conversation with a woman. She is middle-aged, stylishly dressed, African American. Their chairs are angled toward each other, and toward a video camera recording their interaction. Their short and intense conversation – touching on race relations, crime in America, and the anxieties of parenthood – plays out unedited, the recording’s quality somewhat distressed but nevertheless watchable due to its digitization by archivists at Yale University.^1^ If it were not for the woman’s confiding tones, the scene might be mistaken for a television interview on a public access programme, a conversation with the creator of those chilling obedience experiments, the celebrated and controversial Professor Milgram. But this is not a television programme, and the subject is not obedience, or at least not in the way Milgram had previously conceived it. Instead, this video was produced in the course of his pilot studies exploring the ‘cyranic illusion’, a psychological phenomenon that he named after the late 19th-century French play *Cyrano de Bergerac* and its famous balcony scene in which the homely but eloquent Cyrano, replete with an extremely large nose, whispers poetic lines to Christian, a handsome but inarticulate soldier. Together, Cyrano and Christian woo Roxanne, the beautiful and intelligent lady that both men love (Rostand, 1971[1897]).

Indeed, later that day, the woman in the video – let us call her Ms A – converses with strangers, the experimental subjects of Milgram’s studies, in those same chairs and in front of the same camera. Meanwhile, Milgram, hidden away in another room and off screen, tells her exactly what to say through a radio, its small speaker tucked into her ear. To borrow from the drama that Milgram invoked, she is Christian to Milgram’s performance as Cyrano. Milgram provides the intellectual input and thoughts on the one side; Ms A the expression, articulation, and performance on the other. From the video recordings, it appears that she is a willing medium for Milgram’s messages – a ‘cyranoid’, in Milgram’s terminology – though not a preternaturally capable one. In speaking to first one experimental subject and then another, her voice is clipped, slow, and sometimes oddly syncopated, in contrast to her own natural style of communication on display in her earlier conversation with Milgram.

Nevertheless, the cyranic illusion seems to hold. Ms A’s interlocutors betray no sign that they think something amiss. Even when she begins to explain to one subject that she is a cyranoid speaking the words of Dr Milgram, her vocal expression suggests that in this act of debriefing she is still listening to Milgram’s utterances and carefully repeating them. Her interlocutor, the subject, sits still, coming to believe her but slowly. This particular reveal has the frisson of an episode of *Candid Camera* – a popular television show that Milgram openly admired – but it lacks the show’s hilarity (McCarthy, 2009, 2011; Milgram and Sabini, 1979). This was a scientific endeavour, after all, taking place in Milgram’s laboratory at the Graduate Center of the City University of New York (CUNY). And it had more in common with Milgram’s ‘Obedience to Authority’ experiments, and more to say to the ideas of its age, than it may at first appear.

This article revisits this late, largely forgotten project pioneered by Milgram: the cyranoid studies that he pursued from 1977 until his death, from congestive heart failure, in December 1984. Cyranoids, we argue, were opportune creations, reflecting the anxieties and possibilities of the late 1970s and early 1980s, a period of American history characterized by the slow fade of the Cold War, the ascendance of identity politics, broadening distrust in institutions, and the rise to prominence of neoliberal ideology (Brown, 2015; Carroll, 1990; Killen, 2006). Milgram’s cyranoid experiments, though incipient and ultimately incomplete, considered what would happen if one person pretended the mind of another was their own by parroting the words of a secret confederate. Employing an experimental apparatus that included ‘bug-in-ear’ devices, concealed radio receivers, transmitters, and microphones, Milgram found, to his surprise and even jubilation, that his experimental subjects did not seem to notice nor suspect that their interlocutors were relaying the thoughts of a hidden prompter. As Milgram would explain, this was due to the cyranic illusion: our persistent perception that in speaking to another, we are ‘dealing with an autonomously functioning person’ (Milgram, 1992[1984]: 339).

On first look, cyranoids may seem like another experimental ruse, or even gimmick from Milgram, a social psychologist often said to exemplify his field’s penchant for blending dramatics, deception, technology, and scientific authority within its investigations (Bayer, 1998; Brannigan, 1997; Morawski and Donaghue, 2016; Oppenheimer, 2015, 2019). Some of his peers regarded the project as incoherent, half-baked, and superficial. Yet Milgram’s project clearly excited both him and his circle of students at the time. And, in ways distinct from most social psychological experiments, Milgram’s cyranoids have had a curious afterlife, one that Milgram could not have foreseen. After decades of historical obscurity, Milgram’s experimental designs were recently recreated by social psychologists at the London School of Economics, who adapted the experiments to study human responses to artificial intelligence (Corti, 2015; Corti and Gillespie, 2015a, 2015b; Gillespie and Corti, 2016). Artists, designers, philosophers, and media theorists have cited Milgram’s cyranoid apparatus as inspiration for their varied explorations of how current communication and computing technologies influence social interaction and individual psychology (Di Nicola, 2010; McCarthy, 2011; Mitchell, Gillespie, and O’Neill, 2011; Oppenheimer, 2017). Indeed, now, more than 40 years after Milgram hatched his plans for matching varying intellects with disparate visages and voices in a theatrical experimental procedure, his cyranic method has attracted diverse and growing attention. It seems capable of stimulating a host of new thoughts, and exposing prevalent concerns, about identity and social relations in our age of technology-mediated reality and surveillance capitalism. As we perceive it, this revival has less to do with historical or scientific interest in Milgram per se, and more to do with the affordances of cyranoids and the technologies that render them.

But why are cyranoids receiving such attention now, and what might historians of the human sciences learn from this? These questions, we acknowledge, inform the narrative that follows. As historians, our natural starting point for understanding cyranoids – what they might mean and possibly portend – lies in Milgram’s own time and place, and in considering first his intentions and circumstances. To date, surprisingly little attention has been paid to the intellectual sources, immediate context, or authorial descriptions of Milgram’s cyranic studies, despite his work, persona, and influence being a perennial subject of historical and scientific debate (see, for example, Blass, 2000; Brannigan, Nicholson, and Cherry, 2015; De Vos, 2010; Miller, 1986; Nicholson, 2011a, 2011b, 2015; Perry, 2012). Only a few scholars have looked past Milgram’s ‘Obedience to Authority’ studies in the early 1960s to examine the aims, methods, and possible meanings of his later cyranic research. Thomas Blass and Kevin Corti offer complementary accounts that depict in detail Milgram’s failures, suffered amidst his own faltering health, to reach the modicum of intellectual rigour needed to secure funding for his experiments. Meanwhile, his colleagues in social psychology, in the throes of an epistemological crisis, turned further away from his legacy and toward greater scientism (Blass, 2004; Corti, 2015; see also Danziger, 2000; Faye, 2012). Also, significantly for our study, the media scholar Anna McCarthy has delved through Milgram’s archive to find how his Cold War-liberal proclivities informed his making of cyranoids into a ‘twentieth-century philosophical toy’, one that, McCarthy convincingly argues, prefigures certain *neo*liberal assumptions about individual responsibility and authenticity that are now at work in our era (McCarthy, 2011: 192).

Here, in this article, we are concerned not only to further explore what prompted Milgram’s intellectual interest in studying cyranoids. We also ponder the cultural forces and political factors that may have shadowed this enterprise during its brief life, and which may help to account for its current vogue. Admittedly, there is little to be found in Milgram’s archive that directly ties this work of the late 1970s and early 1980s to the political agendas, intellectual debates, or cultural anxieties of the era. However, a close reading of Milgram’s notes on his investigations – including the audiovisual material described above – brings out hints and prompts, veritable voices on and off stage, that intimate how the climate of the time shaped the possibilities of cyranoids for Milgram, his contemporaries, and later interpreters.

To explore this broader context, we draw on recent work on the fraught and unsettled cultural dynamics of the 1970s, notably Daniel T. Rodgers’ intellectual history of the United States during the last quarter of the 20th century, *Age of Fracture* (2011). Rodgers’ account of the 1970s is especially salient for the discussion below. It suggests how, in that decade, earlier notions of social determination were challenged and complicated by renewed interest in individual behavior and historical contingency. Debates that had previously emphasized material economic forces, social norms, and institutions of power were transformed by new ideas about the primacy of the individual and hidden and/or dispersed sources of social authority. That may be too schematic a way to characterize the period, or contrast of periods, for historians of the human sciences accustomed to fine-grained analyses of the changing meanings of ‘the social’ and similar constructs within particular disciplines. Still, in our view, Rodgers helpfully captures something of the self-consciously ‘fractured’ cultural mood of that time. Thus ‘conceptions of human nature’, Rodgers writes, ‘that in the post-World War II era had been thick with context, social circumstance, institutions, and history’ were often refocused in the 1970s and after on problems about ‘choice, agency, performance, and desire’ (Rodgers, 2011: 3). Historians might immediately recognize how by the 1970s, various branches of the human sciences and humanities were investigating an ‘age of fracture’, in the sense that scholars were fascinated less by direct social pressures and more by intersubjective and intertextual processes. The implications of a ‘hermeneutics of suspicion’ (to borrow Paul Ricoeur’s telling phrase) sent many scholars in search of new genealogies of morality, truth, science, gender, and more. Rodgers tracks these shifts and shows more widely how in the same period dominant metaphors of American civic life changed dramatically. These new metaphors – of social fracture, market forces, and individual agency – were reflected in the era’s most influential schools of legal, economic, and political thought, as well as in the observations of journalists, novelists, and cultural critics.

Milgram’s intellectual commitments were forged in the first half of the 20th century, and so reflect that era’s obsession with society and its power to guide and determine individual actions. Yet the ground was shifting beneath Milgram’s feet, in ways well described by Rodgers. We argue that the cyranoid experiments took the questions that had framed ‘Obedience to Authority’ – and had informed Milgram’s research throughout his career – and reformulated them in ways that reflected new intellectual problems, but in a fashion that neither Milgram nor his colleagues could fully recognize. In short, Milgram staged a complex experimental scene comprising ambiguous bodies and voices, some on stage and some invisibly operative off. Whether he intended so or not, the cyranoid apparatus that Milgram invented emerged in a context where the sources and limits of power, authority, representation, and identity appeared increasingly troubling to many observers. Thus, while it can be interpreted in a variety of ways, the cyranoid bears a close relationship with the late 20th century’s prismatic, equivocal, and multiple readings of human relations. The significance of these readings – and their relationship to our current neoliberal order – are still crystallizing, making cyranoids, as a ‘twentieth-century philosophical toy’, more intriguing than ever before, and perhaps only now fully coming into view.

Ultimately, cyranoids are surprisingly good constructs ‘to think with’. Yet why this might matter to historians of the human sciences requires historiographical as well as historical reflection. In our concluding remarks, we draw further on intellectual history, specifically on the historiographical discussions of context, especially its potentially wide-ranging synchronic and diachronic aspects, which featured so notably in arguments about methodology in the very period – the 1970s and 1980s – when the experiments in question took place. Indeed, we are sensitive to how our own methodological impulses – to work with ideas about ‘discourse’ and ‘intertextuality’, and to question the authority of the author over the text – are informed by influential debates in the academy that were contemporaneous with Milgram’s cyranoids. As we hope to suggest, it is perhaps only in the light of 21st-century explorations of that 1970s context, that, as it were *après coup*, a range of latent possible meanings in the original cyranoid project becomes more fully apparent.

## Cyranoids: A late Cold War creation

In all of the cyranic experiments that he undertook, Milgram staged a commonplace social interaction: a conversation or interview. Although his broader research plans involved increasingly intricate situations, involving more than two parties, a basic apparatus would hold: a ‘source’ that was hidden from sight; a ‘medium’ (or ‘cyranoid’) who participated in the conversation by repeating the words of the source; and a naïve experimental subject, whom Milgram called the ‘interactant’. The latter conversed with the medium and was eventually asked to evaluate the medium’s behaviour. There was some mild deception involved, as the interactant would learn the true nature of the experiment only when finally debriefed. This moment of revelation was itself a chance for data collection, to see if subjects would expand upon whether they had found their conversational partner unsettling or implausible in any way. In the pilot studies and formal experiments that Milgram conducted, beginning in 1981, apparently none of the interactants spotted the precise ruse. As Milgram would explain at the 1984 Annual Convention of the American Psychological Association (APA) – his only formal presentation of his cyranoid research – the illusion was so potent that, despite the subject having an opportunity to ask the cyranoid a range of questions, for the subject ‘there is nothing special about the person functioning in the cyranic mode’ (Milgram, 1992[1984]: 339). It was as if the cyranoid, despite its peculiar qualities and complex relays of speech, could pass through normal society like any other individual. In his cyranic studies, Milgram sought to make use of this curious phenomenon for the benefit of science and society.

Below we consider the origins of the cyranoid experiments, and Milgram’s efforts to present them as timeless, rather than timely inventions, as well as socially useful tools. Milgram’s archive, at Yale University, provides many clues to what he and others made of this work, and how the research was situated in, or sought to transcend, a particular time and place. We convey his reflections on the project and draw attention to the significance of the materials he amassed, including videotapes, evaluative responses from colleagues, an equipment catalogue, and more. As Blass and Corti have suggested, Milgram was in fact working it all out, or at least trying to, as he went along. Whether he sought to leave his technique open to as many possibilities of interpretation and application as he could is open to debate. Certainly he viewed the setup as both serious and playful. He was clearly both intrigued and puzzled by what his experiment ‘meant’, or what might be concluded, or even hypothesized, about it. This was partly what exasperated those colleagues who had to adjudicate on his funding applications. They wondered if he had thought through adequately or really knew what on earth he was doing (Blass, 2004; Corti, 2015). However, we are also arguing that various ‘voices off’ – influences and developments perhaps beyond Milgram’s view, or at least his stated intentions – also had a significant bearing on the emergence of cyranoids, and added to their pregnant sense of possibility. We will return to that question later, but first, our aim is to situate cyranoids in the larger discursive frameworks of that era, and to invite discussion of what constitutes their relevant, synchronic, historical context.

### An origin story

According to Milgram’s biographer, Thomas Blass, the idea for the cyranoid studies emerged in an exercise that Milgram posed to students in a course on mass media at CUNY in fall 1977 (Blass, 2004: 239). Students tested whether one person could function as a medium for the words of another by playing the roles of sources, cyranoids, and interactants in a scenario inspired, as noted, by *Cyrano de Bergerac*. In these classroom trials, the students were aware of the setup from the beginning; there were no unsuspecting interactants to deceive with such illusions. Rather, the exercise was to determine whether a source and medium, using a small radio receiver, transmitter, earphones, and microphones, could banter with an interactant as if in normal conversation.

Reflecting on the exercise in his personal notebook, Milgram wrote that the technique was ‘spectacularly effective’, in that the medium appeared to carry on a very natural dialogue, although he was merely tracking the words of the source (quoted in Blass, 2004: 239). Milgram was not alone in thinking that there might be something to the cyranic apparatus that would yield findings beyond the mundane and obvious. Reports written by his students describe the experience as eerie, even uncanny. Some were fascinated by how cyranoids combined the identities of source and medium. As one student remarked, it seemed as if the cyranoid ‘was like a unique musical instrument…playing the music of another, yet on the instrument recognizably that of his own’.^2^ Milgram, recognizing the potential of the technique for social psychological experiment, began to concoct a research programme that would use this method to investigate, *inter alia*, attitude change, crisis negotiation, and person perception.

It is intriguing how Milgram cleaved to the name of a work of fiction to explain and establish his technique: He flagged ‘Cyrano’ in the title of the project, in the naming of roles (namely, the cyranoid), and in defining the illusion that he sought to explain. Milgram’s use of fiction appears deliberate. He knew well the power of situating a psychological phenomenon in current events and pressing social questions, as most popular accounts of his earlier ‘Obedience to Authority’ experiments claimed that his results explained why so many Germans had participated in the Holocaust, and how Americans could just as easily be led to torture and kill. Though Milgram weathered some criticism for aligning his laboratory-based experiments with the terrors of Nazism, the association had, in his mind, an honest claim on his own thinking and that of his audience (Fermaglich, 2006; Miller, 1986; cf. Nicholson, 2011a, 2015).

And yet, with his cyranic investigations, Milgram proposed a project shaped by a much longer and more ambiguous time frame, or perhaps no time frame at all. He seemed to reach for some ageless social drama, suggesting more fundamental human concerns about selfhood, image, and identity. Milgram seemed to want to escape any particular historical context: Edmond Rostand’s play, written during the cultural turmoil of the *fin de siècle*, looked back to an idealized, romantic past – that of the 17th century – and offered a hero who, regardless of the era, epitomized panache in how he disjoined the ugliness of a body from the eloquence and fertility of a mind. Meanwhile, as we have noted, Milgram’s cyranic investigations were devised in an era arguably suffering from its own kind of acute cultural anxieties, even undergoing, as has been suggested elsewhere, something of a collective ‘nervous breakdown’ after the Vietnam War, economic convulsions, and the Watergate scandal (Killen, 2006). Milgram looked to the idealized space of the psychology laboratory and promised a powerful, all-purpose tool for studying the human condition.

Despite its universal themes, *Cyrano de Bergerac* may have been an altogether timely reference for Milgram and his students. Milgram could evidently count on his students and colleagues being familiar with its famous balcony scene. That scene had been recreated several times in film and television shows in the postwar period. The play had recently been revived, thanks to a new English translation by Anthony Burgess (Rostand, 1971[1897]). Burgess was the writer celebrated for *A Clockwork Orange*, a 1960s novel about the abuses of psychological experiment in behavioural ‘re-education’ famously made into a film in 1971 by Stanley Kubrick (Burgess, 1962; Kubrick, 1971). Mere months before *A Clockwork Orange* was first screened in cinemas, Burgess’ adaptation and English translation of *Cyrano de Bergerac* premiered at the Tyrone Guthrie Theater in Minneapolis, Minnesota. The success of this production led Burgess and producer Richard Gregson to reset the play as a Broadway musical, with Burgess contributing the book and lyrics. The 1973 musical, titled *Cyrano*, was a step too far for audiences, and the show closed after 49 performances. Burgess later blamed the failure of the production on ‘union problems’ and the distractions of the Watergate scandal, though he also had his reservations about setting Rostand’s admittedly contrived plot to music. In a 1984 essay, Burgess acknowledged these difficulties, but reminded his readers that the play nevertheless introduced one of the great leading characters of theatre, and that ‘Cyrano has something not altogether superficial to say to an age trying to make a style out of despair’ (Burgess, 1991[1984]: xv).

It is unclear from the available archival evidence why, precisely, Milgram found *Cyrano de Bergerac* so inspiring. It may have been yet another expression of Milgram’s love of theatre, which is already well documented by Blass (2004). Milgram may have even personally met Burgess while the author and translator was teaching full-time at CUNY, as Visiting Professor of English Literature and Creative Writing, during the 1972–1973 academic year, when the *Cyrano* musical was in rehearsal (Biswell, 2005: 348). We cannot be sure. Perhaps, too, Milgram, a well-known Francophile who had lived in Paris, may have been familiar with the original French text written by Rostand.^3^

The effects of drama and fiction on Milgram’s thinking are worth emphasizing. The play provided an implicit touchpoint, a site of meaning to return to, as Milgram’s investigations evolved from classroom exercise to hypothetical research programme to actual and heavily staged scientific experiment. And yet whatever the reference back to a celebrated *fin de siècle* drama, Milgram imbued cyranoids with protean meanings and applications relating to his immediate society, as well as some timeless human problems about deciphering other people’s cues, gestures, and words. First in his proposals to the National Science Foundation, and then in laboratory-based studies, his efforts spoke, intentionally or otherwise, to the emerging intellectual concerns and apprehensions of the 1970s, that is to say, to the disorientated and fractured mood of his time.

### An experimental programme in person perception

Milgram was encouraged by the success of his 1977 classroom exercise to construct a full research programme that would utilize the technique in multiple forms. Foreseeing the need for a large grant, in February 1979 he applied to the National Science Foundation (NSF) programme in Social and Developmental Psychology for $200,000 (Blass, 2004: 239).^4^ In his application, Milgram emphasized that his research was still in an ‘exploratory phase’, in which the parameters of the technique, such as the optimal level of source input, would be investigated alongside topics of perennial psychological interest. As Blass and Corti have argued in their historical accounts of this research programme, Milgram’s proposal was ambitious in scope yet vague in detail, and the specific experiments that he sketched had obvious faults. Not only were there clear ‘confounds’ in his experimental designs that Milgram had not controlled for, but he also left nebulous how these experiments would assimilate or advance the field’s theoretical framework (Blass, 2004: 240; Corti, 2015: 25). If there was a calculated strategy in Milgram’s application, it failed to pay off with his NSF reviewers – both in February 1979, when he first submitted his proposal and was rejected, and in November, when he resubmitted the same proposal with only minor changes. As one reviewer who saw both drafts tersely put it, ‘Its main defect remains a preoccupation with technique over theory’.^5^

Despite its deficiencies, Milgram’s NSF application suggests the daunting array of questions that he hoped his technique could address. And, when read alongside accounts of the experiments that he eventually undertook, his grant application gives some indication of the questions that held the most fascination for him. It appears that of all of the possible, imagined scientific applications of his technique, Milgram was most interested in what cyranoids might reveal about ‘person perception’ – how we perceive others based on our impressions of their appearance, behaviour, personality, and other factors. Milgram’s plans for studying person perception were the most elaborated within his NSF application, and occasioned the greatest number of references to other psychologists’ published papers. And, when Milgram began more formal experiments, it was this particular thread that he followed. Yet while cyranic studies of person perception might have opened a door for timely contributions to the study of how sexism, racism, and other attitudes influenced social judgement – all hot topics in social psychology by the 1970s – Milgram’s efforts were focused primarily, if not exclusively, elsewhere. More important, for him, was understanding the collective psychological mechanisms of interactant, medium, and source that maintained the cyranic illusion. Perhaps because of his own tendency to play the role of the source in these studies, Milgram was most excited by the possibilities afforded to a source who could enter into the persona of another (the medium). He was fascinated, intensely so, by the ‘naivety’, as he put it, of the interactant who failed to notice anything awry.

In his NSF application, Milgram proposed multiple kinds of experiment on person perception. One strand of this research promised to investigate gender bias by pairing a single source first with a male medium and then a female medium, and asking interactants to converse with each and evaluate their performances. To underline the topicality of this method, Milgram cited recent psychological research on whether individuals, of either sex, evaluated a written prompt differently if they knew the sex of the author, or if the text itself was about a man or a woman (Deaux and Taynor, 1973; Frank and Drucker, 1977; Goldberg, 1968; Pheterson, Kiesler, and Goldberg, 1971; Rosen and Jerdee, 1974). There was something static and bloodless about these text-based experiments. Milgram offered his cyranic technique as a methodological breakthrough in studying biases in vivo, in the dynamic give-and-take of an actual conversation between an interactant and a cyranoid. Such experiments, he noted, might also explore the effects of race and age on interactants’ perceptions. Milgram thus gestured at a traditional topic of postwar social psychological research, the study of prejudice, partiality, and attitudes toward difference, but one that, since the 1960s, had taken a reflexive turn, as if psychology’s own theories and methods required reassessment (Herman, 1995; Richards, 1997).

Yet, in his later investigations, Milgram would prioritize perfecting his technique over contributing to theories of person perception. This was the case even as he was at pains to recruit mediums and subjects from miscellaneous ethnic groups, and both sexes, of diverse ages and education levels – markers of group identity that he treated prima facie as indicators of difference for the purposes of creating interesting encounters between cyranoids and naïve subjects. In his first pilot study of 1981, he videotaped subjects speaking with various mediums, including Ms A (the middle-aged black woman described above), a young white woman (perhaps one of Milgram’s students), and a young white man with a streetwise New York accent. The latter Milgram described as a ‘shoe-shine boy’, whose lack of education, Milgram later remarked, led him to mis-repeat that ‘Plato was a great falafeler’, a distortion of ‘philosopher’ (Milgram, 1992[1984]: 344). As Anna McCarthy glosses these later investigations, Milgram was interested in the ‘burlesque possibilities’ of his technique, even ‘finding humor in the collision of different sex, age, educational, and socioeconomic backgrounds that resulted’ (McCarthy, 2011: 196). In the video recording of Ms A acting as medium, Milgram repeatedly has her speak French to a subject – a journalist from France – although she stumbles over its pronunciation.

Milgram himself performed the role of the source in this pilot study and the ensuing experiments, and he seems to have assessed the eeriness of the cyranoid in each of these instances by how far the medium diverged from his own identity as a white, male, middle-aged, Harvard-trained professor. Indeed, in his NSF proposal he described a second series of person perception experiments that would push the limits of the illusion by testing whether extreme dichotomies between source and medium would be recognized, and under what conditions. Milgram asked, ‘What degree of disparity between medium and source will be perceived as falling within the range of normalcy? [What would] lead to the perception of anomaly, and how are such anomalies explained?’^6^ He took up this agenda in formal experiments in 1983–4 after receiving a small grant of $5000 from CUNY to study cyranoids with the assistance of undergraduates in his experimental social psychology course. With this modest support, Milgram and his students conducted ‘Experiment 1’, with Milgram as source, 20 unsuspecting subjects, and four mediums: Ken, a 16-year-old black male high school student; Jay, a 16-year-old high school student of Korean origin; Christine, a 22-year-old female graduate student; and Stuart, a 32-year-old male graduate student (Milgram, 1992[1984]: 339). Here, each subject interviewed one cyranoid, asking a pre-arranged list of questions on personal and political subjects, the latter mainly about nuclear disarmament. After each interview, the subject was asked to rate the medium on maturity, poise, intelligence, likability, and sincerity.

The subject was also invited to evaluate the truth or falsity of certain statements about the medium, including whether the medium ‘spoke by receiving radio messages and repeating them to me’. In reporting the results of this experiment at the 1984 APA convention, Milgram chose his words carefully. None of the 20 subjects had thought that their conversation partners were receiving instructions by radio. Some had offered glowing assessments of their mediums, and felt pangs of loss upon learning that the person they had spoken to did not exist as such (Milgram, 1992[1984]: 339–40). But Milgram did not mention that eight of the subjects had some suspicion that the medium was not another experimental subject, but probably a stooge or confederate of the experimenters.^7^ The so-called cyranic illusion, at a pinch, could simply be one’s failure to imagine that an interlocutor was enacting the enduring paranoid fantasy of being controlled by radio waves.

The results of Experiment 1 were promising enough that in early 1984, Milgram and his students conducted ‘Condition 2’. This more elaborate experiment involved hiring two young boys – Jason, 11 years old, and Omri, 12 years old – from a nearby acting school, and training them as mediums with Milgram as their source. A panel of high school teachers served as interactants, tasked with evaluating Jason and Omri on their intellect – to see if, truth be told, they could tell that a 50-year-old Harvard-trained professor was discernible in the boys’ unusually gifted responses. The boys’ cyranic performances were so capable that no interlocutor questioned whether they were speaking their own thoughts. Milgram observed that this may have been due to the teachers’ preconceived notions about what were fair questions to ask the boys. Milgram confessed, ‘As the source, I was hoping they would ask the cyranoid about Freud, Jung, Adler, or at least Darwin and Wittgenstein, but some teachers stuck to fractions and parts of speech’ (Milgram, 1992[1984]: 343).

Milgram boasted of the possibilities of cyranoids for illuminating the mechanisms of person perception, including the gendered, racial, ageist, and other biases that one often relies on to stitch together impressions of another person into a semblance of integrated selfhood. Yet in actual practice, Milgram was most interested, as mentioned, in the productive possibilities of the illusion, its essential quality being the discrepancy between source and medium. He thus leveraged contemporary concerns about group differences and their role in constructing identity in order to give significance to an emergent and, so he hoped, beneficent new social technology: the cyranoid. Read one way, the whole undertaking might contribute to calling such social categories into question, helping to render sexism, racism, ageism, and other -isms obsolete, like the human-machine cyborgs of Donna Haraway’s 1985 feminist manifesto (Haraway, 1985). Haraway, in her now famous essay, presented the figure of the cyborg, the cybernetic fusion of human and machine, as a catalyst for rethinking feminist-socialist politics. Perhaps the cyranoid, like the cyborg, might prove the inadequacies of judging someone’s thoughts, and other political attributes, by her socio-biological appearance.

A more sceptical reading, offered by McCarthy, suggests how these hybrids – by combining certain sources with a diverse array of mediums – could be enrolled in the incipiently neoliberal project of arming traditionally powerful individuals with new capabilities for acting untrammelled, and according to their own desires, within an increasingly hollowed-out society (McCarthy, 2011: 193). It is telling that Milgram, in his 1984 remarks, compares the cyranic source to the wizard, Frank Morgan, in *The Wizard of Oz*. These sources, Milgram explained, ‘remain poised in another room eagerly spewing their words into a microphone and listening attentively to the proceedings so that they can feed their lines to the cyranoid on cue’ (Milgram, 1992[1984]: 337). Sources might have their own insecurities and personal limitations – and Milgram certainly did – but with the help of this particular setup, they could project power and authority. Thus, however much Milgram gestured to contemporary concerns about bias and prejudice against marginalized groups in society, he was also invoking a new technology of surveillance and infiltration.

### Old technologies, new affordances

Though later artists and psychologists have sometimes interpreted Milgram’s cyranoids as tools for exploring the *cutting edge* of artificial intelligence and human–computer interaction, the technologies Milgram relied on were more quotidian and already obtainable at that time in the shopping mall, or at least via specialist stores. From the beginning, Milgram relied on widely available commercial electronics to achieve the illusion of seamless conversation. He demonstrated these devices, and their arrangement in his laboratory, in a video that he included with his NSF application.^8^ The video, composed from material recorded in December 1977, shows students playing the roles of subjects and mediums, with Milgram serving as source. In the first part of the video, we observe a male medium sitting across a table from a female graduate student, and we hear them discuss her research project. The video helpfully zooms in on the medium, and the viewer can see that he is fitted with a bug-in-ear form of device, a wire curling around the back of his neck. Then the video cuts to Milgram sitting in an adjacent control room, watching the students’ conversation through a one-way mirror. The female interactant’s voice can be heard speaking, and Milgram periodically leans toward a microphone to instruct the medium in how to respond. There is a noticeable doubling of the conversation as we see Milgram express the very words that we have just heard from the medium. Milgram, in a voice-over narration, explains, ‘The echoing effect that you’ll see and hear in the control room is the actual tracking of the medium’. Consequently, because of video editing, we observe how a seemingly bilateral conversation actually involves cyranic triangulation. Later in the video, the radio receiver is revealed: The female student has become the medium, and she is talking to a younger woman, who is unaware of taking part in a test of the cyranic illusion. The subject does not believe it when the medium explains the technique, and so, with a little *coup de théâtre*, the medium produces the radio, which has been in her lap under the table, and she pulls out the earpiece. The medium hands them to the interactant so that she, too, can hear the voice of Dr Milgram in her ear.

As Milgram himself admitted, his use of these devices was not new to psychologists.^9^ Since the 1950s, clinical psychologists had experimented with bug-in-ear radio devices for training their students in how to work with clients (Kroner and Brown, 1952). Similar studies in the early 1970s made use of affordable electronic components from Radio Shack – transistor radios, miniature microphones, and in-ear speakers – in the training of therapists and, in clinical experiments, in instructing mothers in how to coax, comfort, or discipline their children constructively (Boylston and Tuma, 1972; Gordon, 1975). In these latter experiments, mothers were observed by psychologists as they interacted with their children. The psychologists offered advice and prompts to the women, but not lines to be repeated word for word (Gordon and Kogan, 1975; Wimberger and Kogan, 1974).^10^

Yet, while these components were affordable, widely available, and well known to psychologists in the late 1970s, their potential uses had been expanding in ever new directions since mid-century, finding application in hearing aids, personal entertainment, military communication, surveillance technology, and more (Curtis, 1976; Mills, 2011; Schiffer, 1991). By the end of the 1970s, miniature radio transmitters in the form of bugs and wires were being used by detectives in undercover sting operations and domestic intelligence gathering on criminals; they were no longer the sole provenance of spies and foreign intelligence agents (Marx, 1988). Interestingly, Milgram’s archive includes a catalogue from Law Enforcements Associates, a New Jersey-based company that sold high quality electronic aids to law enforcement and private investigators. The catalogue advertises, for $295, a set of miniature wireless induction earphones. The product description explains their multiple uses:

These wireless receivers…can cue actors on stage, provide private communication to one person in a meeting or crowd, serve to communicate with roving tour guides, and perform many other one-to-one communications functions. In security and law enforcement, these small wireless earphones can permit a plain clothesman to utilize two way radio without a tell-tale earphone cord.^11^

It cannot be assumed that Milgram, with his minimal research budget, necessarily purchased these earphones or other equipment advertised in the catalogue. Perhaps he simply fantasized about doing so, and about how they might render cyranoids able to move freely within and beyond the laboratory.

It is intriguing to recall how security and surveillance became a leitmotif of American popular culture in the years after the Watergate scandal, sounding again and again throughout the 1970s and after. In 1974, Francis Ford Coppola’s film *The Conversation* introduced many Americans to a growing subculture of private surveillance experts and their miniature devices for wiretapping and secretly recording private communications (Coppola, 1974). The film’s famous opening sequence, in which a couple’s conversation is surreptitiously recorded as they walk through San Francisco’s Union Square, demonstrated the capabilities of shotgun and parabolic microphones, multitrack sound mixing equipment, and also – in a contraption worn by a police detective turned private surveillance expert – a hidden microphone attached to a small tape recorder and earpiece. Coppola’s production was long in the making, but its filming happened to parallel the senate hearings that familiarized Americans with the Nixon campaign’s efforts to bug the Democratic National Committee headquarters in the Watergate office complex, and Nixon’s secret tape recordings of White House conversations with their curious erasures.^12^ When the film premiered, *The Conversation* seemed to many moviegoers strikingly topical in offering a vision of a brave new world of technologically advanced eavesdropping, and the paranoia and guilt it wrought on individuals caught in the web of surveillance. Also noted was the way in which the film suggested how radically mistaken any audience might be when following a putative plot or listening to a seemingly innocent snatch of conversation; motives, circumstances, and plans were not necessarily what they might seem to the eavesdroppers.

Milgram’s cyranoid can be interpreted as an avatar of surveillance, that is to say, of surveillance as it was understood in this period. It was an officer on the front lines of a sting operation: Repeating lines from a ‘central intelligence’, cyranic mediums were supposed to ingratiate themselves with subjects, offering themselves as persons to be interpreted when, in reality, it was the subject’s thoughts and feelings that were dissected in Milgram’s experiments. Indeed, there was also another piece of equipment used in these studies, not essential to the technique but nevertheless important: the video camera that, by recording the subject and medium talking, documented the cyranic illusion at work. The camera was not hidden, and subjects gave their permission to be recorded; hence they knew they would be watched. They were simply unaware that they were being observed and addressed by a hidden individual, a secret agent, who – in Milgram’s particular experimental design – knew much more about what was going on, and what precisely was being investigated, than the subject ever could. What Milgram perhaps did not intuit was that the subjects might also have thoughts, silently held back, that the ‘source’ and experimental interpreter never really apprehended. The subjects might, for instance, knowingly be playing their own part, that of the ‘naïve’ interlocutor. If the experiment explored or dramatized subterfuge and secret interferences, it could also be read as a symbol of hermeneutic uncertainty. Be that as it may, Milgram’s cyranoid studies took his subjects’ ready familiarity with surveillance as tacit approval to try out new methods of infiltration, monitoring, and disruption, and then to see what actually transpired.

Steeped as they were in that particular era, Milgram’s cyranoids can also inspire new debate about surveillance and the ambiguities of interpretation in our own time, hence the notable revival of interest in this work. The artist and design historian Maya Oppenheimer, for example, has lately explored how Milgram’s final studies might be updated, and monetized, for the needs of surveillance capitalism. In a performance piece, she offers a sales pitch for a ‘range of imagined, “deceptive” devices’ that read gesture and voice intonation against a massive databank of profiles scraped from YouTube videos, security camera footage, and other sources using artificial intelligence. She explains, ‘Essentially it’s an AI-informed personality reader that uses the Cyrano idea but flips it for what would be a commercially viable security tool: to ascertain whether an individual’s embodied performance is real, and they mean what they say’. At the end of her sales pitch, Oppenheimer reveals that she is an artist, not a security expert. She has simply imagined the present-day applications of Milgram’s cyranic ideas, and constructed an archive of historical documentation, for a fictional corporation, to match.^13^ That she might be taken seriously as having a viable commercial vision raises, to be sure, a number of troubling questions.

### Cyranic persuasion and negotiation

Cyrano, the wizard of Oz: These were the fictional renderings of the cyranoid that Milgram used to describe his research agenda. His scientific undertaking, we suggest, was bound up in theatre, fiction, television, and cinema in more ways than one. Further, as we have just discussed, Milgram instilled his apparatus with possibilities for invisible infiltration, manipulation, and the projection of authority at a distance. This was not accidental, though Milgram may not always have been forthcoming in describing cyranoids’ full range of powers and applications. Previous historical accounts have noted how Milgram privately questioned the scientific value of cyranoids, yet meanwhile, his mind raced with possibilities for how his apparatus might be applied to real-world situations, for instance in matching a physically disabled and verbally talented salesperson with an abled body, the better to sell products door to door (Blass, 2004: 242–3; Corti, 2015: 21–2, 28; McCarthy, 2011: 192). Whatever the commercial, political, and theatrical spin-offs, Milgram, no doubt seeking to retain the required academic gravitas in his NSF application, chose to focus on two particular capabilities of cyranoids that, once perfected, might take them beyond the laboratory: persuasion and negotiation. He detailed two distinct threads of research that he would pursue, in addition to his studies of person perception, that would make these capabilities manifest, and of serious worth. Of course, Milgram never did conduct these investigations, perhaps because he was unsuccessful in his grant application or, more likely, because he ran out of time. In these abandoned studies, Milgram invited his colleagues to imagine, with varying degrees of conviction, future plausible worlds where real-life cyranoids, armed with skills in persuasion and negotiation, might flourish outside of the laboratory.

To study ‘cyranic persuasion’, Milgram suggested in his NSF application that he would investigate how audiences reacted to a lecture given by a cyranoid, and then compare these responses to a control case in which a lecture was given by the source directly. Milgram, by then a seasoned public speaker as well as university lecturer, probably saw inherent value in studying how verbal and nonverbal communication combined to affect persuasive ability. However, he did not offer much precision in his experimental designs. As one NSF panellist queried, why use the cyranoid apparatus at all when the lecturer could memorize a script written by the source?^14^ Another reviewer, nonetheless, recognized the potential payoff in placing a hospitable face on technical information, noting ‘this is both *a fascinating and scary bit of technology*—think of taking Economics I with Gregory Peck as the medium and Milton Friedman or J. Kenneth Galbraith (depending on your point of view) as the source’.^15^ It is worth pausing over this reviewer’s specific reference to economics, that is, to the heated public battle between neoliberal, microeconomic analysis and the older Keynesian, macroeconomic school. In 1979–80, when Milgram’s proposal was under evaluation, this debate was taking place not only within universities but in the halls of government, in courts, between think tanks, and on the pages of the *Wall Street Journal* and other newspapers – even on the public television channel PBS, which broadcast Milton and Rose Friedman’s *Free to Choose* in 1980, a direct response to Galbraith’s 1976 television series *The Age of Uncertainty* (Rodgers, 2011: 50–73). Consequently, Milgram’s ‘fascinating and scary bit of technology’ hinted at the potential for not only dressing up technical material for undergraduates, but also smuggling in controversial ideologies in the warm tones and welcoming address of a polished Hollywood actor.

Indeed, Ronald Reagan would achieve just this feat in November 1980 with his election to the presidency. Aided by gifted speechwriters like Peggy Noonan, Tony Dolan, and Peter Robinson, Reagan might himself be viewed as a cyranoid presence, repeating lines and projecting authority as he acclimated Americans to a new political rhetoric of optimistic individualism (Rodgers, 2011: 15–40). However, his apparatus of choice was not the in-ear radio but the teleprompter, an invention of the 1950s that quickly became a tool (and often a crutch) for politicians, television personalities, newsreaders, and anyone else who had to speak correctly yet ‘authentically’ to mass audiences. As McCarthy suggests (2011), the teleprompter is essentially a cyranic device. She reminds us that such technologies are surprisingly ubiquitous:

Technologies like the teleprompter and the earpiece, allowing the speech acts of one individual to originate in the mind of another are a standard means for organizing consciousness and controlling interpersonal interactions on television, providing seemingly neutral and practical solutions to the problems that impromptu speech can cause in diverse arenas of television production. (ibid.: 186)

The teleprompter and the earpiece, McCarthy argues, have the same psychological effect, namely, the construction of an ambiguous, or perhaps even an unseemly individual, whose authenticity appears both diminished and enhanced by technology. Put another way, the cyranoid enacts a sort of benevolent intelligence on stage, apparently situated within one person, but in reality achieved through a distributed system of off-stage labour.

In his proposed study of cyranic *negotiation*, Milgram imagined applying the cyranic technique to the sort of emergency counselling situation that can arise when police are called to a scene to mediate with a hostage taker or someone threatening to commit suicide. What if the officers responding to the incident could tap a psychological expert, in a remote location, who might instruct or even script them? Milgram explained that this study would test ‘the bargaining or negotiating skill that can be imparted to the medium through the cyranic method’.^16^ This potential application may have spoken to Milgram with the urgency of the era. In 1972, the New York Police Department, in response to the hijacking and murder of the Israeli Olympic team in Munich, created the first of what became an increasingly common phenomenon thereafter: a ‘Hostage Negotiation Team’. In its formation, the team had the input of a clinical psychologist-cum-detective, Harvey Schlossberg, who developed strategies for engaging hostage takers without violence (Gelb, 1977; Schlossberg, 1980). Though the NYPD Hostage Negotiation Team was spared any copycat incidents of international terrorism, they routinely responded to persons threatening suicide, barricading themselves, or taking hostages in the course of robberies or hijackings.

Milgram’s exploration of cyranic negotiation absorbed the anxieties and assumptions of its time in still another way. In 1973, after a spate of violent robberies and hijacking incidents, the journalist Tom Wolfe declared in *Esquire* that hostage-taking was the ‘ideal crime’ of the decade. That year, a robbery in Stockholm had given name to Stockholm Syndrome, the phenomenon whereby hostages fall in love with their captors and come to identify with their causes (Wolfe, 1976[1973]). The most high-profile victim of Stockholm Syndrome in the 1970s was Patty Hearst. Her story of the good-girl-gone-radical was splashed across newspapers throughout the decade. The heiress, kidnapped by the violent Leftist group the Symbionese Liberation Army (SLA) in 1974, was on the lam through the summer of 1975, stood trial in late 1975, was found guilty in 1976, and was released from prison in 1979 after her sentence was commuted by President Jimmy Carter (Graebner, 2008; Killen, 2006: 261–73). The American public was entranced by how, in tape-recorded statements released by her captors, Hearst had advocated the radical revolutionary positions of the SLA and declared that she had not been ‘brainwashed’, but had willingly undergone a personal, political transformation. Many Americans wondered whether she was speaking her own thoughts – whether she was still the same ‘Patty’ at all, or merely the mouthpiece of others – and whether she deserved sympathy or condemnation. Consequently, if hostages in the 1970s could appear as cyranoid-like dummies, ventriloquizing for their captors, Milgram suggested that police could negotiate with the puppetmasters by becoming cyranoids themselves.

### Age of fracture

The last 25 years of the 20th century might be cast as an age of fracture in American intellectual history, as Rodgers explains, not because Americans resisted unifying narratives or shared metaphors, but because the narratives and metaphors themselves celebrated or at least concerned fragmentated states. Talk of social reality was often displaced by discussions of irreducibly personal and partial – individual – experiences. It was also characterized by a focus on micro-circumstances, market behaviour, and the capacity of language and culture to call into being choices and alternatives (Rodgers, 2011).

Moreover, Milgram’s experiments were contemporaneous with explosive, disruptive discussions within academia – for instance, about false consciousness or illusions surrounding such assumed figures as the author of the text. Think here of Barthes, Derrida, Kristeva, and Foucault, for example, so many of whose works were translated, read, and applied in the American academy in the 1970s, not least at Yale, which was a notable centre of engagement with deconstruction. Not far in the future lay a plethora of new explorations of how identity is linked to and complicated by language and performance; consider, for example, the interventions of Judith Butler that disrupted categories of gender and much besides. The decades that followed brought not only a radical questioning of assumptions about innateness, fixity, and settled identity, but also new inquiries into hybridity (Donna Haraway) and everyday forms of intersectionality (Kimberlé Williams Crenshaw). These developments did not pass unnoticed by social psychologists, particularly those interested in the social meanings of gender, race, sexuality, and other markers of identity. We do not need to strain the evidence by placing Milgram’s late experiments in the realm of post-structuralism, postmodern philosophy, or other critical studies. We merely note the turbulent and interrogative intellectual mood of the time, in which prior ways of seeing, listening, and interpreting were so extensively contested. It was a time when the fraught relationship between authors and texts, creators and audiences, or even, as Milgram might put it, sources, mediums, and interactants, came to the fore.

In this period, the problem, and the opportunity, that Milgram identified was the ease with which one can be covertly persuaded, egged on perhaps, by one’s superficial assumptions about others. His experiments revealed how most people will easily assume, in conversation, that they are engaging with other autonomous people who are, in turn, speaking their minds. Milgram considered that such assumptions could be put to excellent social uses: to explore pressing questions in psychological research, but also to fashion – with hidden and helpful ‘words in the ear’ – better workers, police officers, therapists, negotiators, or parents. The cyranoid illusion, he proposed, could make certain kinds of interactions more efficient, effective, and fruitful. The trick was to separate physical presence from intellectual expertise, body from mind, text from author. But his project also suggested grounds for concern about how easily we might take advantage of these new abilities to control, coerce, and ventriloquize. Thus, even as Milgram’s investigations at CUNY seemed to resonate with the incipient anxieties of the late Cold War period, they also captured the Reagan era’s optimism by insisting the experiment was a social and economic opportunity, a chance for new forms of civic and entrepreneurial activity.

## Obedience to cyranoids

Our emphasis thus far has been on novelty and inventiveness. But the cyranoid studies can also be interpreted as continuous with the earlier endeavours that made Milgram famous: the ‘Obedience to Authority’ experiments conducted in New Haven in the 1960s. These earlier studies evoked a storm of controversy over, among other things, the proper, ethical relationship between the psychological experimenter (and his stand-in, the experimental apparatus) and the human subjects of research. Milgram’s plans for his cyranic investigations triggered a similar discussion, though it was much smaller in scale and, importantly, focused on the relationship between the experimenter’s confederates, the source, and the medium, who must work together to create the cyranic illusion. As with his obedience experiments, Milgram’s questionable assumptions (or after-the-fact rationalizations) about human resilience and individual agency can explain his failure to anticipate criticism (Nicholson, 2011a, 2011b; Stark, 2010).

In the 1970s, the Obedience experiments, though long concluded, were not far from Milgram’s thoughts. It was only in 1974 that he had published his monograph *Obedience to Authority: An Experimental View.* In 1977, he put out a collection of his papers and essays, *The Individual in a Social World*, a section of which also dealt with the Obedience experiments. According to Blass, during these years, he was making good money in speaker fees, and giving frequent invited talks about Obedience (Blass, 2004: 231–2). The massacre at My Lai and the mass suicide at Jonestown, revelations of state-sanctioned torture in Latin America, and other high-profile calamitous events of the late 1960s and 1970s contrived to make Milgram’s contributions to psychology perpetually *au courant*. His Obedience experiments also continued to be critiqued within academic circles for their possible ethical failings, even as they became fixtures of psychology textbooks (Stam, Lubek, and Radtke, 1998). Milgram was frustrated by how he had been cast as a careless or cavalier researcher in these debates, and how that enduring sense of controversy over his ethics brought his results, as well as his bona fides, into question. Milgram’s response to these continuing discussions was fully engaged and defensive, even in the 1970s (Blass, 2004: 235–8).

There are hints that Milgram saw continuity between his two projects, and that his contemporaries were inclined to agree, though for different reasons. In his discussion of the general psychological significance of his cyranic research programme, found in his NSF application, Milgram suggested that his cyranoid studies were explorations of social influence, a theme that he had often said, in various writings and interviews, was his abiding intellectual interest (Milgram, 1977; Tavris, 1974). He explained that social influence was evident in these new studies in two essential ways: the cyranoid’s influence on the interactant’s perceptions, and the source’s influence on the medium.^17^ Milgram aimed to explore the former thoroughly and systematically in an experimental research programme; here was social influence as it related to person perception, persuasion, and negotiation. The latter, the source’s influence on the medium, would not be so central to his agenda, despite his provocative hints otherwise in his NSF application. Though Milgram did not acknowledge this outright, there were potential ethical issues to studying the source’s influence on the medium, issues akin to those he had encountered in his Obedience to Authority experiments. Though he only alluded to this possibility in his NSF application, some of his proposal’s evaluators noted an omission.

According to Milgram, there had to be agreement and coordination, indeed an explicit pact, between the source and the medium for the cyranic apparatus to work effectively. The medium would enter this relationship knowingly and voluntarily, subordinating his or her own identity to that of the source – or, more properly, to a hybrid identity – for the duration of the experiment. Milgram acknowledged the possibility that a source might abuse this position of influence, for instance in asking the medium to make utterances that the medium did not agree with or found uncomfortable to say, or in leading the medium to provoke a negative reaction in the interactant. Sources might thus take advantage of being able to speak to a stranger without placing their own body in harm’s way, to act without direct consequences to their own reputation or safety. Milgram would later call this the ‘disinhibition hypothesis’ (Milgram, 1992[1984]: 344). In his NSF application, however, he described this phenomenon as deindividuation, and aligned it with what Philip Zimbardo, in his widely famous Stanford Prison Experiment, and Milgram too, in his Obedience experiments, had studied previously. Yet deindividuation, in the cyranic vein, not only freed sources from the consequences of their actions; it also allowed them to watch another person take the brunt. It is worth quoting Milgram at length:

The cyranic mode entails a separation of the person’s utterances from the broader processes of attitude, judgment, and feeling that is the hallmark of the integrated individual. It thus raises questions of responsibility and individual accountability. In this respect the cyranic mode bears on questions of deindividuation discussed by Zimbardo (1973) and others (Milgram 1974). In Zimbardo’s experiments, individuals wore masks and then carried out aggressive acts. He found an increase in aggression when the individuals could not be identified. The cyranic mode goes a step further in that the individual who is originating the verbal statement does not merely have a mask; he has another individual to absorb and appear responsible. We are thus dealing with a phenomenon that goes beyond anonymity, in that the responsibility for the assertions made by the source are deflected onto another person. The socio-psychological questions become whether the source will be affected by this arrangement, and make assertions which he would not have made if the responsibility were focused on him.^18^

Milgram reveals here his fascination for the source’s relationship with the medium, and its potential to precipitate dramatic psychological events within the laboratory. His in-text references were of course to Zimbardo’s prominent public account of the Stanford Prison Experiment, ‘The Mind is a Formidable Jailer’, published in the *New York Times Magazine* in April 1973, and Milgram’s own monograph, the capstone of his earlier research, *Obedience to Authority: An Experimental View*. For Milgram, these were gold-standard social psychological experiments. For some of Milgram’s readers, there could be no other references more evocative of the controversial turn that social psychology had recently taken in its treatment of human subjects.

Despite the criticisms surrounding his and Zimbardo’s work, Milgram did not find the influence of the source on the medium to be an ethical dilemma in the offing. He noted that the source’s control over the medium was ‘superficial’ because ‘the cyranic mode entails control of the verbal output of the person without first acting on deeper or intermediate levels of personality’. He therefore declared that this was not akin to covert manipulation, and compared the source’s influence on the medium to how learning a language affects a person’s ‘decisional space’, shaping their fundamental view of the world but giving them the power to question, dissent, persuade others, and generally exhibit agency as an individual.^19^ Agency was key: the medium, in his view, was always technically free to break his or her pact with the source by refusing to relay, or deciding to modify, the utterances of the source. Milgram had displayed a similar notion of human agency in how he had treated the experimental subjects of his Obedience experiments, putting them in a situation designed to coerce their obedience but rationalizing that they always possessed the mental freedom to question the setting and their role within it (see, for example, Milgram, 1964).

Unsurprisingly, not all of Milgram’s NSF reviewers shared his sanguine belief in the resilience of the medium. Jerome Bruner, Milgram’s mentor from Harvard and the one reviewer who put his name on his comments, wrote that ‘the “medium” who transmits the messages of the “source” is bound to experience the effect of messages that he transmits that are not his own.…I would strongly urge that Professor Milgram include in his proposal some way of monitoring such possible effects’.^20^ Another reviewer asked,

But what effect can the situation have on the source and the medium? (For example, if the medium delivers an argument in which he doesn’t believe, or speaks more or speaks less skillfully than he is able, or interacts in a manner generated by the other sex or a social class not his own?) This question also contributes an ethical issue, and I would urge both on theoretical and ethical grounds that it be investigated.^21^

Milgram found that other colleagues at CUNY had similar reservations. When he first submitted his proposal to the NSF, he provided a copy of the same proposal to CUNY’s Committee on Human Subjects, which would need to approve his plans before any actual experimentation could take place. In his applications to the NSF and CUNY, Milgram focused his discussion of experimental ethics on the use of deception, a traditional element of social psychological experimentation that some critics had felt he pushed too far in his earlier work (see, for instance, Baumrind, 1964; Mixon, 1972; Stark, 2010). Milgram argued that the deception of the cyranic experiments was altogether mild and unharmful, as it stemmed from the ‘misrepresentation of the medium as being the source of his own verbal output’.^22^ Yet his evaluators were not so concerned with deception, but with the medium’s freedoms, real or otherwise, to choose not to comply with the source. The CUNY committee approved Milgram’s proposed research, subject to conditions that effectively ruled out any experiments, at least in the near term, on the source’s experience of deindividuation or disinhibition.

Questions about compliance to authority of one kind or another run like red thread through much of Milgram’s oeuvre. His Cyrano work, even in its diminished and sanitized form, extended and responded to ideas he had already developed in his earlier inquiries, as well as the critical accounts these had generated. In this new venture, he hoped, the experimenters could study the point (if any) at which we might come to doubt the body–mind relationship of our interlocutors, the capacity (or failure) to perceive and act upon the knowledge at some point of an implausible quality in the other’s communication. Thus, the experiment would reveal if the volunteer could put two and two together, noting some delay, hesitation, oddity, or eccentricity in the interlocutor’s speech patterns that might suggest a mismatch between the person doing the articulating and the origin of the words that they were mouthing. Could the subject get interested, he seemed to be asking, in who was responsible for these words? If they could not even notice, or perhaps even if they did, then ‘Cyrano’ could be put to new uses. This ingenious experiment, in other words, enables us to consider not so much the subject’s own obedience directly, but how far the subject can detect that another is acting passively under instruction, rather than thinking for themselves.

## Cyranoids in context

In our attempt to recover the historical meanings of Milgram’s cyranoid experiments, we have asked what were the intellectual and social circumstances of the 1970s and early 1980s, and even the legacies of the 1950s and 1960s, that can shine explanatory light on the experiments and their afterlives in the present day. Yet, given the relative sparsity of source material and the undeniable open-endedness of these investigations – which seem to invite questions about the meanings of interpretation, context, illusion, and more – we also want to invite debate about what a satisfying and sufficient historical account of this work would look like. Given the evident limitations of Milgram’s own investigations and articulations of cyranoids, a close reading of his archive is not sufficient. Yet at what point do we risk, as historians, putting words into Milgram’s mouth, ideas into his text, or thoughts into his mind that simply do not belong there? Faced, in other words, with a flexible and diffuse explicandum, we want to highlight the problem of what it means to consider the cyranic studies historically, and thus reflect on our historiographical practices and methods of inquiry. We seek here, in other words, to place cyranoids in context, but also to use this set of experiments to reflect upon what context means, and where it might end. Because there is much that remains enigmatic and provocative about his venture, we have found Milgram’s project a surprisingly useful tool to think with, as well as an intriguing case to think about.

The periodization of Milgram’s cyranoid experiments places additional demands on our historical endeavour, raising questions about what our own methods and concerns may have in common with Milgram’s intuitions. Indeed, the 1970s and early 1980s, the years in which Milgram’s last experimental adventure were dreamt up, were also a period, as we have noted, in which the relation of text and context was widely explored and problematized. Do our own historiographical impulses – which, as is the case for many historians, owe so much to the linguistic and cultural turns of the 1970s and 1980s – share something of the epistemic regime that shadows Milgram’s later work (however diffusely)?

Within the historical profession, the 1970s and 1980s saw a marked interest in, and expansion of, strategies for considering historical context and rethinking the aims of interpretation. These strategies, both new and old, were not without controversy. For example, readers of this journal will be familiar with how debates about ‘social construction’ were, and continue to be, fraught for historians of science. We do not seek to revisit those debates here. For the purposes of this discussion, let us take a rather different and equally notable example, elsewhere in the field of intellectual history, of how new interpretations of context were probed, particularly to expand the bounds of its synchronic and diachronic dimensions. By chance, the year of Milgram’s death marked the inauguration of a major new series of books, ‘Ideas in Context’, edited by Quentin Skinner, Richard Rorty, and Jerome B. Schneewind. This series was committed to thinking historically about languages of politics and to producing books that, as its prospectus announced, ‘discuss the emergence of intellectual traditions’ (Rorty, Schneewind, and Skinner, 1984). This series consolidated certain innovations in intellectual history made during the previous 20 years; it promised a history not of ideas, as previously understood, but of the procedures, aims, and vocabularies in which ideas had taken shape, competed with alternatives, and found adoption by varied audiences. To paraphrase Skinner, the new Cambridge school was interested not so much in what authors and texts meant, but in what they were ‘doing’ in certain times and places.

A year later, in 1985, as volume two of this series, J. G. A. Pocock’s *Virtue, Commerce and History: Essays on Political Thought and History, Chiefly in the Eighteenth Century* discussed Skinner’s contributions in developing the field, first by exploring intention and context, second by considering ever more closely the question of the speech act itself and what it was doing. Yet Pocock found himself reaching not just for the synchronic dimension that Skinner had done so much to elucidate in his *Foundations of Modern Political Thought* (1978) but also, more ‘futurologically’ perhaps, to explore a diachronic dimension, to pay heed while interpreting work in the past to the future that lay in front of it. As Pocock had demonstrated in *The Machiavellian Moment* (1975), the tracking of Niccolò Machiavelli’s intellectual context might take historians back far into the past and even into an as yet unknown set of developments, before and beyond Machiavelli’s own lifetime. Thus, in 1985, Pocock offered a rich account of the possible limits, or perhaps the potentially limitless nature, of ‘context’. He mused here, with a Hegelian flourish, on how ‘self knowledge is retrospective, and every author is his own owl of Minerva’ (Pocock, 1985: 4). The text constructed the meaning we believed the author might have intended, but an author might only know it afterwards, perhaps, through the very act of writing – or perhaps the author might never quite know it at all. Pocock observed how the more complex, even the more contradictory, the language context in which a given actor in history was situated, ‘the richer and more ambivalent become the speech acts’, and ‘the greater becomes the likelihood that these acts will perform upon the context itself and induce modification and change within it’ (ibid.: 5). He wondered whether we could assume that an actor in history could know what they actually thought before saying it, and thus whether we could recover intentions before they were articulated in a text – from another text, that is, or even from texts as yet unborn.

Pocock (1985: 6) mused on the gap between ‘intention and effect, or between consciousness of the effect and the effect in itself’, thus asking about discursive or other actions performed in an open-ended time, an open-ended series of effects, that start to stretch out the meaning of their context. Consequently, if we asked, *à la* Skinner, *what is the author (or the text) ‘doing’ here?*, an ever more protean range of answers was possible, given this as yet unborn potential of meaning, in the author’s own act of composing and in future readings and conditions to follow. The work might be ‘doing something’ that entirely escaped not only the author’s consciousness but also their moment in time. This invited the dizzying thought that certain ‘contexts’ might only be apparent long after the author’s death. Pocock suggested here, in remarks clearly informed by the post-structuralist thoughts of that time, that it was not clear whether an author’s or text’s action was ever over and done with. The recovery of authorial intention vied with the multitudinous series of possible unforeseen meanings and possible posthumous ‘actions’ generated by the ‘play’ of the text, which was anything but the possession of its author.

We propose that this perspective, so strikingly set out during the 1980s within intellectual history, is relevant to our study of Milgram’s cyranoids. Our article situates an episode from the past within its synchronic context. Yet we also seek to pick up traces of interest and anxiety in that time that were not by any means fully articulated. Our account is shadowed, admittedly, by our own concerns and apprehension of neoliberalism, systems of surveillance, and technologies of artificial intelligence and virtual presence. How far this was something Milgram’s experiment was already investigating, or indeed intimating, and how far it is a context that was simply not there, but yet to come, is open to question. We invite the reader to consider how far the evidence supports the claim that such concerns were already latent in, or prefigured by, Milgram’s project. We are wary of ‘teleological readings’, yet also aware that these later ‘contexts’ might be precisely what help us to see certain features of the experiment and its surrounding discourse, *après coup* as it were. We invite debate about how far our attention to such resonances may enrich, or distort, the appropriate historical interpretation of this particular experiment’s meaning in its own time and place.

Ultimately, there is a tension in Milgram’s efforts with cyranoids. We see him at points seeking to determine, even control, the meanings of this work: to perfect an illusion, to revel in mastery of a technique, and to reassert the ethics of his experiment. Inside his laboratory, often it was he who gave words and direction to the cyranoid and archived the resulting performance. And yet his investigations were marked or, as critics at the time thought, marred by a certain looseness, even vagueness, as well as a kind of ludic possibility. He allowed (to put it generously) others some rein to interpret and reinterpret what he *was doing with* cyranoids, and what more could be done with his apparatus, then and since. Subsequent artists, designers, theorists, and psychologists have found in these hybrids glimpses of neoliberal monsters, human avatars of artificial intelligence, and more. They, like we, have noted in his experiment worrying signs of our deracinated globalized times, saturated in new forms of surveillance and subterfuge. The cyranoid, concocted in an age of fracture, is now a 21st-century being, acting as a catalyst for further experiment, debate, and performance. In its multiplicity, its many voices on and off stage, it is even now perhaps signalling dangers and opportunities for interpersonal relations and social communications in the decades to come, albeit in ways we cannot foretell.

## Notes

Our thanks to Sarah Marks, Charlie Williams, Ian Magor, Katie Joice, and the anonymous HHS readers of our article for their helpful comments and suggestions. We are also grateful to staff at Yale University library, especially Stephen Ross, Manager of Public Services, Manuscripts and Archives, for their assistance.

1.
*Cyranic Discussions* [Video tape] (1981) Stanley Milgram Papers (SMP), Manuscripts and Archives, Yale University, MS 1406, Box 26.3U.

2. Anonymous students quoted in Stanley Milgram, ‘The Technique of Mediated Speech as a Tool in Social Psychology’ (application to National Science Foundation), 1979, SMP, Box 24, Folder 12, pp. 13–14.

3. It should be noted that Milgram does not cite Burgess’ English translation of Rostand’s play in his National Science Foundation application and APA talk, but Brian Hooker’s 1923 translation.

4. This would translate into about $730,000 today.

5. Anonymous reviewer, ‘Proposal Evaluation Form’, n. d., SMP, Box 24, Folder 15.

6. Milgram, ‘The Technique of Mediated Speech as a Tool in Social Psychology’, p. 10.

7. Data analysis, 1983, SMP, Box 25, Folder 18.

8.
*Cyranic Speech: Pilot Studies* [Video tape] (1977) SMP, Box 26.2U.

9. Milgram, ‘The Technique of Mediated Speech as a Tool in Social Psychology’, p. 6.

10 For a review of psychological studies using the apparatus that Milgram describes, contemporaneous with Milgram’s cyranic research, see Schwitzgebel and Taylor (1980).

11. Law Enforcement Associates catalogue, ca. 1977, SMP, Box 25, Folder 21.

12. Coppola had written the script during the 1960s. He was inspired by Antonioni’s 1966 film about paranoia and photography, *Blow Up*, to create a film about eavesdropping and the new technologies of sound recording that were then altering film-making as well as the worlds of police and private surveillance (Coppola, 2011).

13. Personal communication with Maya Oppenheimer, October 2018.

14. Anonymous reviewer, ‘Proposal Evaluation Form’. See also Blass (2004: 240).

15. Anonymous reviewer, ‘Proposal Evaluation Form’; emphasis added.

16. Milgram, ‘The Technique of Mediated Speech as a Tool in Social Psychology’, p. 9.

17. Ibid., pp. 12–14.

18. Ibid., p. 14.

19. Ibid., p. 12.

20. Jerome Bruner, ‘Proposal Evaluation Form’, n. d., SMP, Box 24, Folder 15, p. 2.

21. Anonymous reviewer, ‘Proposal Evaluation Form’, n. d., SMP, Box 24, Folder 15.

22. Milgram, ‘The Technique of Mediated Speech as a Tool in Social Psychology’, p. 15.
